# Predictors of No-Shows After an Endoscopic Mucosal Resection in Veterans: A Retrospective Analysis

**DOI:** 10.7759/cureus.76462

**Published:** 2024-12-27

**Authors:** Mahmoud Y Madi, Yassine Kilani, Hayden Rotramel, Michelle Baliss, Jill Elwing, Gregory Sayuk, Ahmad Bazarbashi

**Affiliations:** 1 Gastroenterology, Saint Louis University School of Medicine, Saint Louis, USA; 2 Internal Medicine, Saint Louis University School of Medicine, Saint Louis, USA; 3 Gastroenterology and Hepatology, Washington University School of Medicine, Saint Louis, USA; 4 Medicine, Washington University School of Medicine, Saint Louis, USA; 5 Gastroenterology and Hepatology, Saint Louis Veterans Affairs Medical Center, Saint Louis, USA; 6 Advanced Endoscopy, Washington University in Saint Louis, Saint Louis, USA

**Keywords:** colonoscopy adherence rate, colonoscopy and polypectomy, conventional endoscopic mucosal resection (cmer), no-show rates, patient follow-up

## Abstract

Introduction

Endoscopic mucosal resection (EMR) is a common intervention for large colorectal polyps, but its long-term success depends heavily on post-procedure surveillance to detect recurrence. Despite the critical importance of follow-up appointments, some patients fail to attend these crucial visits. This study aims to identify demographic, clinical, and socioeconomic factors that predict missed follow-up appointments after EMR. By understanding which patients are at the highest risk for non-compliance, we can develop targeted strategies to improve follow-up adherence and optimize long-term outcomes in this population.

Methods

We conducted a single-center retrospective study at the Saint Louis Veterans Affairs Healthcare System of patients presenting for colon polyp EMR between January 2019 and December 2022. The primary outcome was the no-show rates for the scheduled patient endoscopic follow-up among patients who underwent EMR. A predictive model was then constructed to yield adjusted odds ratios and their prospective 95% confidence intervals.

Results

A total of 69 patients met the inclusion criteria. Fifty-nine patients followed up within the recommended interval. Ten patients failed to attend their follow-up appointments and were labeled as no-shows. The predictors of no-shows with their adjusted odds ratios and 95% confidence intervals in descending order included previous history of one or more no-shows (3.8, 1.7-8.5, p = 0.002), recommended follow-up interval greater than six months (3.5, 1.1-12.5, p = 0.04), psychiatric comorbidities (2.8, 1.7-4.6, p = 0.002), and history of substance use (2.7, 1.8-6, p = 0.03).

Conclusions

In patients undergoing EMR, our study identified several risk factors for follow-up non-compliance, including previous no-show history, substance use disorder, and psychiatric comorbidities. These findings suggest that targeted interventions for high-risk patients may improve follow-up adherence. While longer follow-up intervals (>6 months) were associated with increased no-show rates, modifying established surveillance guidelines would require dedicated studies evaluating the safety and efficacy of alternative follow-up schedules.

## Introduction

With a projected 152,810 new cases and 53,010 deaths in the United States (U.S.) in 2024, colorectal cancer (CRC) remains a major oncological burden in the U.S., ranking third in both incidence and cancer-related mortality among both genders [1]. While the overall CRC incidence has declined by 1.8% per year since 2011-2012 in both men and women, a notable increase in cases among individuals under 55 years old comprising about 20% of new cases in 2019 highlights persistent prevention challenges [1]. CRC has now emerged as the leading cause of cancer-related mortality in men younger than 50 years and the second leading cause of cancer mortality in women younger than 50 years [2].

Numerous studies have shown that CRC screening coupled with the detection and removal of precancerous colon polyps can effectively lower the incidence and mortality of CRC by 50%-60% [3-7]. Endoscopic mucosal resection (EMR) has emerged as the standard of care for the management of large non-pedunculated colorectal polyps (LNPCPs) measuring 20 mm or greater [8-10]. However, vigilant post-EMR surveillance is crucial due to the potential for recurrence [10,11]. Recommended follow-up intervals post-EMR depend on the size and removal method of the polyps, as well as the polyp histology. Generally, shorter follow-up intervals are needed for larger polyps, piecemeal resections, and cases where high-risk precancerous features or early cancer are found [12]. The United States Preventive Services Task Force (USPSTF) guidelines recommend a three-colonoscopy surveillance regimen for piecemeal resections of adenomas or sessile serrated lesions ≥20 mm at six months, one year, and three years post-EMR [12]. Despite complete EMR, recurrence rates of adenoma range from 15% to 30% [13]. Follow-up colonoscopy can be challenging for patients due to bowel prep requirements and potential logistical challenges (e.g., the need for a driver to accompany the patient and missed work), and as a result, some patients do not show up for their planned post-EMR surveillance colonoscopy [14]. However, there is a paucity of literature outlining the predictors for “no-show” to follow-up visits post-EMR.

Therefore, to further advance the current knowledge and improve follow-up post-EMR, we conducted a single-center retrospective study from January 2019 to December 2022 exploring differences in baseline characteristics of patients undergoing post-EMR follow-up and the predictors of no-shows. By identifying high-risk populations prone to no-shows, we aim to implement strategic interventions to enhance the overall adherence to post-EMR follow-up protocols.

An abstract version of this manuscript was previously presented as a meeting poster at the 2024 ACG Annual Meeting on October 27, 2024.

## Materials and methods

Data source

Using the Saint Louis Veterans Affairs (VA) Healthcare System (STLVAHCS), we conducted a single-center retrospective study of patients who underwent EMR from January 2019 to December 2022. We identified patients who underwent colorectal EMR through electronic medical health records from the VA healthcare system and then tracked their subsequent visits within the VA healthcare system. The medical records retrieved were then de-identified. This study was approved by the STLVAHCS Associate Chief of Staff for Research and the Education Review Board as a quality improvement initiative.

Study population

Consecutive English-speaking individuals ≥18 years old who presented for follow-up of colorectal polyp EMR from 2019 to 2022 were included in the analysis. Patients with a history of CRC or inflammatory bowel disease were excluded. An outpatient follow-up EMR was defined as an outpatient visit in which a follow-up EMR was listed as a primary diagnosis. To capture this, we retrospectively reviewed all the cases completed by the advanced endoscopists working at the VA center during the study period. We reviewed the STLVAHCS electronic medical records of endoscopy to determine whether patients followed up as scheduled for post-EMR endoscopy. Those who did not present for the endoscopic follow-up as scheduled were labeled as “no-shows.”

Patient and hospital characteristics

We captured data available within the STLVAHCS on age, sex, race (White, African American, and others), and comorbidities including obesity, diabetes mellitus, coronary artery disease, antiplatelet or anticoagulant use, family history of CRC, and personal history of advanced adenoma.

Outcomes

The primary outcome was the no-show rate for patients who underwent EMR. We assessed potential predictors for no-shows including the American Society of Anesthesiologists (ASA) class, EMR follow-up >6 months, veteran transportation services, history of advanced adenoma, antiplatelet or anticoagulant use, substance use disorder, psychiatric comorbidity, previous one or more no-shows to appointments within one year of the study date, primary care physician within the VA, EMR Boston bowel preparation score, EMR total number of polyps, polyp pathology, and interval clinic follow-up.

Statistical analysis

Categorical variables are presented as counts and percentages; continuous variables are presented as means with standard deviation. A logistic regression model was constructed encompassing patients’ demographics, comorbidities, and procedure-related factors to yield adjusted odds ratios (aOR) and their prospective 95% confidence intervals (CI). Statistical analyses were performed using the Statistical Package for the Social Sciences (SPSS) 27.0 (IBM Corp., Armonk, NY, USA).

## Results

Baseline characteristics

A total of 69 (N = 69) patients (mean age 66.8 ± 7 years, 8.7% female patients, 18.8% African American) met the inclusion criteria of our study. Table 1 summarizes the baseline characteristics of the included population. Fifty-nine patients (85.5%) followed up within the recommended interval after the initial EMR. Only 10 patients (14.5%) failed to attend their follow-up appointment and accordingly were labeled as no-shows. When comparing baseline EMR pathology between the follow-up and no-show groups, we found tubular adenomas (67% vs. 50%), hyperplastic polyps (0% vs. 20%), tubulovillous adenomas (16.9% vs. 20%), sessile serrated adenomas (11.8% vs. 10%), and adenocarcinoma (3.4% vs. 0%). While these differences were not statistically significant (p = 0.6), it is important to note that patients with adenocarcinoma were referred for surgical evaluation and appropriate cancer treatment based on staging, with colonoscopic surveillance resumed only after the completion of cancer therapy. When compared to individuals who underwent follow-up, no-shows were more likely to be male patients (100% vs. 90%), non-Hispanic Whites (90% vs. 80%), obese (70% vs. 56%), and patients with a family history of CRC (30% vs. 5%) (Table 1).

**Table 1:** Baseline characteristics of included patients. CRC: colorectal cancer

Characteristic	Followed up (N = 59)	No-show (N = 10)
Age (mean, standard deviation)	66.8 ± 6.7	66.7 ± 9.4
Sex (N, percentage)		
Male	53 (89.9%)	10 (100%)
Female	6 (100%)	0
Race (N, percentage)		
White	47 (79.7%)	9 (90%)
African American	12 (20.3%)	1 (10%)
Obesity (N, percentage)	33 (55.9%)	7 (70%)
Diabetes (N, percentage)	27 (45.8%)	3 (30%)
Coronary artery disease (N, percentage)	17 (28.8%)	3 (30%)
Antiplatelet or anticoagulant use (N, percentage)	18 (38.5%)	4 (40%)
Family history of CRC (N, percentage)	3 (5.1%)	3 (30%)

Predictors of no-show

The predictors of no-shows included a previous history of one or more no-shows (aOR = 3.8, 95% CI: 1.7-8.5, p = 0.002), recommended follow-up interval greater than six months (95% CI: 3.5, 95% CI: 1.1-12.5, p = 0.04), the presence of psychiatric comorbidities (aOR = 2.8, 95% CI: 1.7-4.6, p = 0.002), and history of substance use (aOR = 2.7, 95% CI: 1.8-6, p = 0.03). The use of veteran transportation services showed a trend to decrease the rates of no-shows without reaching statistical significance (7% vs. 14.5%, p = 0.06). The results are summarized in Table 2. Other factors associated with no significant difference in follow-up rates include the ASA class, the EMR Boston bowel preparation score, antiplatelet or anticoagulant use, history of advanced adenoma, the total number of polyps resected, the presence of a primary care physician (PCP) within the VA, and the presence interval clinic follow-up (p = 0.61).

**Table 2:** Predictors of no-show and definitions of variables analyzed. ASA: American Society of Anesthesiologists; EMR: endoscopic mucosal resection; aOR: adjusted odds ratio; CI: confidence interval; VAMC: Veterans Affairs Medical Center *: if applicable; -: not applicable; +: adenomas that are 1 cm or larger, with tubulovillous or villous histology, or exhibiting high-grade dysplasia

Predictor	p-value	aOR*	95% CI (lower, upper)*
ASA class	0.113	-	-
EMR follow-up >6 months	0.04	3.5	1.1-12.5
Veteran transportation services	0.06	-	-
History of advanced adenoma^+^	0.12	-	-
Antiplatelet or anticoagulant use	0.79	-	-
Substance use disorder	0.03	2.7	1.8-6
Psychiatric comorbidity	0.002	2.8	1.7-4.6
Previous one or more no-shows	0.002	3.8	1.7-8.5
Primary care physician within the VAMC	0.56	-	-
EMR Boston bowel preparation score	0.96	-	-
EMR total number of polyps	0.88	-	-
Interval clinic follow-up	0.61	-	-

## Discussion

Our study shows that several patient factors are linked to increased rates of no-shows among veterans scheduled for EMR follow-up in the VA healthcare system. Predictors identified included previous history of no-show and recurrent no-show, recommended follow-up interval greater than six months, history of substance use disorder, and psychiatric comorbidities. Surprisingly, patients with a family history of CRC were more likely to not show up. There was no significant difference in follow-up rates across ASA classes and EMR Boston bowel preparation scores and related to a history of antiplatelet or anticoagulant use, a history of advanced adenoma, the total number of polyps resected, the presence of a PCP within the VA, and the presence interval clinic follow-up and VA transportation services.

EMR is currently the standard of care for the resection of LNPCPs [8-10]. One meta-analysis showed that local recurrence after EMR of non-pedunculated colorectal lesions is 15%, with lower local recurrence rates (3%) for en bloc resections compared to piecemeal resections (20%) [13]. Notably, over 90% of recurrences are detected within six months post-EMR [13]. These findings support the implementation of targeted surveillance strategies based on resection methods and emphasize the importance of complete en bloc resection whenever feasible to minimize recurrence risk.

Follow-up can be challenging for patients, particularly due to the need for repeat bowel preparation, and may be difficult for patients with limited resources, transportation, and special support networks [14]. Our study showed a trend toward improvement in compliance when utilizing veteran transportation services without achieving statistical significance. This is likely due to a lack of power to show a significant difference. Such finding allows future studies to explore the impact of supervised transportation on compliance rates. This study is one of the first and few studies evaluating the rate and predictors of no-shows [15,16]. When compared to individuals who underwent follow-up, no-shows were more likely to be male patients, non-Hispanic Whites, obese, and patients with a family history of CRC. One larger retrospective study found that the African American race was associated with higher rates of no-shows in gastroenterology endoscopy [15]. The findings of this study highlight several important predictors of patient no-shows, expressed in terms of odds. A prior history of no-shows was associated with 3.8 times greater odds of missing appointments (p = 0.002). Similarly, patients with recommended follow-up intervals exceeding six months had 3.5 times greater odds of a no-show (p = 0.04). Psychiatric comorbidities were also a significant predictor, corresponding to 2.8 times greater odds of no-show (p = 0.002). Finally, a history of substance use was linked to 2.7 times greater odds of no-show (p = 0.03).

While our study is the first to identify predictors for no-shows for patients post-EMR, other authors performed similar studies for patients undergoing surveillance colonoscopy. Shuja et al. found that patients scheduled for surveillance colonoscopy had better adherence than initial screening [15]. Furthermore, studies have shown that the implementation of simple measures (e.g., telephone calls and message reminders) was effective in reducing the non-attendance rates in endoscopy [16,17]. Therefore, given the high recurrence rates of adenoma post-complete EMR, additional efforts should be targeted toward simple and personalized interventions to reduce the likelihood of no-shows and ensure a timely follow-up to reduce rates of progression to CRC [13].

This study has several strengths. This is the first study that assesses the characteristics of patients labeled as no-shows post-EMR, to improve adherence to the recommended follow-up post-EMR. Furthermore, our analysis included a comprehensive overview of possible predictors for post-EMR visit no-shows, including patient-specific and hospital-specific predictors. We also note several study limitations. The single-center and retrospective nature of our study, coupled with our limited sample size to a unique population of U.S. veterans, may limit the external validity and generalizability of our findings. However, addressing these potential biases can be achieved through further prospective multicenter studies and meta-analyses.

## Conclusions

In summary, for patients undergoing EMR, previous history of no-shows, recommended follow-up interval greater than six months, history of substance use disorder, and psychiatric comorbidities were linked to increased rates of no-shows. While these identified risk factors suggest potential benefits from enhanced attention and support services (like transportation and communication resources), modification of established follow-up guidelines would require validation through larger prospective clinical trials. Special attention to these high-risk patient factors may help improve compliance rates within current practice frameworks.
